# Hot Water as an Obtundent

**Published:** 1904-02-15

**Authors:** S. H. Guilford


### HOT WATER AS AN OBTUNDENT.

BY S. H. GUILFORD, D.D.S., PH. D.

The dental profession has run the gamut of pain obtundents from sharp instruments, creosote and zinc chloride to warm air, cataphoresis and various medicinal compounds. Of all these, dehydration of the dentin seemed to be the method that gave the best results and the one that could most generally be depended upon.

The electric current alone availed little, while in cataphoresis the current was used only to carry the cocaine or other drug deeper into the tooth structure. By depriving the dentin of its moisture one of the main essentials for the transmission of sensation was removed. To be informed now that an excess of moisture, when raised to a high temperature, will accomplish the same result reverses our previous ideas upon this subject.

Hot water has been used for years by certain practitioners to lessen or obviate the pain ordinarily attendant upon the grinding of the crowns of natural teeth previous to the mounting of crowns and bridges, but its employment for the obtunding of pain in excavating has for the first time been revealed to the writer through an appliance devised for this purpose by a Mr. Shoenberg, of California.

Our attention has been called to it by Dr. Frank L. Platt, of San Franciso, who says that he has had one of the appliances in constant use for several months, and is enthusiastic in his praise of it and the results it has yielded. In its original form there was a cup or receptacle which held about a half pint of water which was heated to the boiling point by a gas jet. Through this cup there passed a copper worm of small diameter which was connected with the public water supply. A small rubber tube extended from the free end of the worm to a fine metal tube, which was attachable to the engine handpiece by meaus of spring clips. The metal tube contained a plug or cut-off which was under the control of the forefinger, thus allowing the stream of water to flow rapidly or slowly at will. The outer opening of the metal tube was no larger in diameter than an ordinary toilet pin, thus supplying a jet that could be applied to the smallest cavity. In operation, the boiling water in the main receptacle heated the water that passed through the copper worm, heating it more when it passed slowly and less when it passed quickly.

The rapidity of the flow and, therefore, the degree of heat was regulated by the little valve in the outlet tube under finger control.

In the improved device the electric current is used to heat the copper coil direct and the appliance is so much reduced in size that it can be attached to the rim of a fountain spittoon, where the water connection is readily made with the worm.

In use, the water jet is directed into the cavity and regulated by finger pressure until the highest degree of heat that the tooth will stand is obtained, after which the stream and the bur operate together, to the great comfort of the patient. When preferred, the jet tube can be detached from the handpiece and directed by an assistant.

As the water accumulates in the mouth it can be carried off by a saliva ejector, or the operation can be suspended from time to time to allow the patient to discharge it into the spittoon. It can be used equally well with the rubber-dam in position by simply depressing the dam so as to form a pocket next to the tooth (in the lower jaw), and carrying off the accumulation with the saliva ejector.

In the upper jaw napkins or towels can be used to receive and absorb the water flowing from the tooth. Besides its principal function, the water jet clears the cavity of the cuttings and keeps the bur free as well.

Although devised principally for use in the preparation of cavities, it serves an equal purpose in relieving pain while while grinding down teeth for the adjustment of crowns and bridges.

One of its advantages is its simplicity and convenience, while another is the saving of time. Under former methods —whether medicaments, warm air, or cataphoresis were used—considerable time had to be consumed before the tooth was in a proper condition to be operated upon.

With the hot water method, excavation can be begun almost as soon as the jet is applied.

It is not claimed, I believe, that the method will entirely desensitize a cavity or tooth surface in all cases, but it does in many, and in others it produces a marked diminution of sensation. This is as much as could truthfully be claimed for any previous obtundent.

That the results are real and not imaginary has been proven by blindfolding a dentist-patient and noting the effect of the instrument when the water was turned on and when it was not.

If further experiment with this new method should confirm previous results, it would seem that we have at hand something which we have all been anxiously awaiting, for every means tending to lessen pain in operations upon the teeth are welcomed alike by patient and operator.

A facetious friend recently observed that for relieving pain, or even for reassuring a patient, he saw no reason why hot water should not be infinitely preferable to "hot air."—*Stomatologist.*

